# Cardiac Involvement in Children Affected by COVID-19: Clinical Features and Diagnosis

**DOI:** 10.3390/diagnostics13010120

**Published:** 2022-12-30

**Authors:** Elena Vasichkina, Daria Alekseeva, Vadim Karev, Ekaterina Podyacheva, Igor Kudryavtsev, Anzhela Glushkova, Anastasia Y. Starshinova, Dmitry Kudlay, Anna Starshinova

**Affiliations:** 1 Almazov National Medical Research Centre, St. Petersburg 197341, Russia; 2 H. Turner National Medical Research Center for Children’s Orthopedics and Trauma Surgery of the Ministry of Health of the Russian Federation, St. Petersburg 196603, Russia; 3 Children’s Clinical Research Center for Infectious Diseases, St. Petersburg 194100, Russia; 4 Institute of Experimental Medicine, St. Petersburg 197376, Russia; 5 V.M. Bekhterev National Research Medical Center for Psychiatry and Neurology, St. Petersburg 192019, Russia; 6 Medical Department, Saint Petersburg State Pediatric Medical University, St. Petersburg 194100, Russia; 7 Pharmacology Department, I.M. Sechenov First Moscow State Medical University, Moscow 119992, Russia; 8 Institute of Immunology, Moscow 115522, Russia

**Keywords:** Myocarditis, children, coronavirus infection, COVID-19, SARS-CoV-2, cardiovascular inflammation

## Abstract

COVID-19 (*Coronavirus disease 2019*) in children is usually mild. However, multiple organ disorders associated with SARS-CoV-2 (*severe acute respiratory syndrome-related coronavirus 2*) have been detected with poor respiratory symptoms. Cardiac changes are noted in 17% to 75% of cases, which are associated with diagnostic difficulties in high-risk groups for the development of complications that are associated with myocardial damage by the SARS-CoV-2 virus. The objective of this review is to identify the most significant symptoms of cardiac involvement affected by COVID-19, which require in-depth examination. The authors analyzed publications from December 2019 to the October 2022, which were published in accessible local and international databases. According to the analysis data, the main sign of myocardial involvement was increasing as cardiomarkers in the patient’s blood, in particular troponin I or troponin T. Many authors noted that the increased level of CRP (C-reactive protein) and NT-proBNP, which are accompanied by changes in the ECG (electrocardiogram) and EchoCG (echocardiography), as a rule, were nonspecific. However, the identified cardiac functional dysfunctions affected by SARS-CoV-2, required an cardiac MRI. The lack of timely diagnosis of myocardial involvements, especially in children at high risk for the development of complications associated with SARS-CoV-2 myocardial injury, can lead to death. The direct damage of the structural elements of myocardial blood vessels in patients with severe hypoxic changes resulted from respiratory failure caused by SARS-CoV-2 lung damage, with the development of severe acute diffuse alveolar damage and cell-mediated immune response and myocardial involvement affected by SARS-CoV-2 damage. In this article, the authors introduce a clinical case of a child who dead from inflammatory myocardities with COVID-19 in a background of congenital heart disease and T-cell immunodeficiency.

## 1. Introduction

The new infectious disease COVID-19 made the whole world shudder because of a serious global threat to the health of the planet’s population, on the one hand, and, on the other hand, it activated the study of new pathological conditions, immunological changes associated with the development of this infection and the influence of concomitant pathology on its course, and the need for searchingnew methods of diagnosis and treatment infectious consequences [1,2].

In February 2020, WHO (World Health Organization) gave the official name for this disease—COVID-19 (*Coronavirus Disease 2019*)—and in 2021 declared COVID-19 a global pandemic, the consequences of which by December 2022 led to 633.0 million confirmed cases of the disease and about 6.0 million deaths (https://www.who.int/emergencies/diseases/novel-coronavirus-2019, 30 November 2022).

Today, the variability of the course of COVID-19 from an asymptomatic to a severe course of the disease with the development of acute respiratory failure, septic shock, and multiple organ damage with a possible fatal outcome is known [3,4].

In about 80% of cases, the disease proceeds in a mild form with the development of symptoms characteristic of a viral infection, which are very diverse and are characterized by the appearance of temperature, fatigue, sore throat, rhinitis, cough, and decreased sense of smell to anosmia [5]. Against the background of the described respiratory symptoms, multiple organ disorders, including those of the heart, are increasingly being recorded in both adults and children [6,7].

Possible manifestations of myocardial damage in children are myocarditis, heart failure, arrhythmias, etc. [8].

Mechanisms of myocardial dysfunction development in MIS-C are not fully understood. Some scientists consider that the reason for damage in the myocardium in adults is hypoxic and ischemic damage, caused by damage to the coronary microvessels or coronary artery disease, acute myocarditis, the syndrome of a systemic inflammatory reaction [6]. From the viewpoint of pathophysiology, probably, an acute infection leads to acute cardiac injury and after that to the development of a post-viral immunological reaction and systemic hyperinflammation, which leads to the emergence of inflammation and dysfunction of the myocardium in predisposed individuals [7].

According to some studies, it has been demonstrated that the main morphological manifestation of myocardial damage in COVID-19 is so-called endothelitis with dysplasia and activation of endotheliocytes, leading to hemorrhages, thrombosis of intramural arteries, and necrosis [6,8].

It is known that extensive organ damage occurs with COVID-19, which is explained by the expression of ACE2 (*Angiotensin-converting enzyme 2*). SARS-CoV-2 can enter cells expressing ACE2 but not cells without ACE2 or cells expressing other coronavirus receptors, such as aminopeptidase N and dipeptidyl peptidase 4 (DPP4), suggesting that ACE2 is the cellular receptor for SARS-CoV-2 [9,10].

Viruses associated with cell-free phagocytosis and macrophages can spread from the lungs to other organs with the high expression of ACE2 through the circulation. Moreover, ACE2 is highly expressed on myocardial cells, renal proximal tubule cells, and bladder urothelial cells [8]. The presence of a long-term damaging viral agent, hyperinflammatory reactions, and a decrease in ACE2 expression leads to the disruption of recovery processes [9].

Among the possible of myocardial damage causes the increased level of cytokines and an immune-inflammatory response were detected, the direct invasion of the SARS-CoV-2 virus itself into cardiomyocytes, pulmonary insufficiency, and hypoxia leading to oxidative stress and damage to myocardial cells [11,12].

At present, the pathophysiological mechanism of myocardial injury in SARS-CoV-2 infection is not completely known, despite the increased number of publications on cardiac involvement in the pathological process in the pediatric population [13].

The aim of this review was to identify the most significant symptoms and methods in the diagnosis of myocardities by COVID-19.

### The Methods of Review

We analyzed publications for the period December 2019 to October 2022, published in accessible international databases (“Medline”, “PubMed”, “Scopus”), for the keywords “myocarditis”, “children”, “cardiovascular inflammation”, “COVID-19”, “SARS-CoV-2”, “severe acute respiratory syndrome coronavirus 2”.

## 2. Features of Diagnosing Cardiac Involvement Affected by COVID-19

The most common symptoms of the cardiac involvement in children with COVID-19 are chest pain or tightness, palpitations, or hypotension up to shock syndrome [11,14,15]. In addition, children with myocarditis associated with SARS-CoV-2 had polymorphic skin rash, conjunctival effusion, and pain in the head and abdomen [16].

According to the literature, in patients with acute myocarditis associated with SARS-CoV-2, higher values of C-reactive protein and the level of pro-B-type N-terminal natriuretic peptide (NT-proBNP) are compared with acute myocarditis not associated with SARS-CoV-2. It should be noted that acute myocardial injury is accompanied by an increase in the level of troponin I/T, creatine kinase MB (CK-MB), and lactate dehydrogenase [6,11,12,17,18].

Based on the results of a retrospective cohort study, the potential role of troponin I levels in predicting heart damage in COVID-19 in children and the need for further examination by a pediatric cardiologist was shown [19]. Furthermore, researchers studying myocardial damage against the background of MIS-C reported that, in addition to troponin I, NT-proBNP can also be used for the early diagnosis of heart disease in a pathological process in children with COVID-19 [11,20].

A special group of patients is children with congenital heart defects, cardiac arrhythmias, heart failure, hereditary cardiomyopathies, and other heart diseases, since the course and consequences of COVID-19 can be much more severe in them due to the development of severe cardiovascular disorders [21].

Cardiac disorders are successfully diagnosed using electrocardiography (ECG) and echocardiography (ECHOCG); if indicated, the magnetic resonance imaging of the heart (MRI) and computed tomography (CT) can be used [16].

Analysis of electrocardiographic changes has shown that sinus tachycardia; T-wave inversion; ST-segment anomaly; heart axis deviation to the right; prolongation of the corrected QT interval; various arrhythmias and conduction disturbances, including sinus arrest, can be recorded in COVID-19 [16,22,23]. In adolescents, according to ECG data against the background of COVID-19, tachycardia was diagnosed, as was, in rare cases, bradycardia [18,22,24].

Echocardiography can detect serious structural anomalies of the heart, such as ventricular dysfunction, dysfunction of the valvular apparatus, dilatation, aneurysm or ectasia of the coronary arteries, dilatation of the heart chambers, and pericardial effusion [25]. Thus, the most common finding according to echocardiography is myocardial dysfunction, which, according to various authors, occurs in 30% to 100% of cases. Various anomalies of the coronary arteries in MIS-C (*multisystem inflammatory syndrome in children*) are observed in 9–75% of cases [26,27,28].

ECHOCG is the main available tool for identifying various structural and valvular anomalies of the heart with an assessment of its function [29]. However, the sensitivity of ECG and echocardiography in diagnosing myocardial disorders due to COVID-19 is significantly lower than MRI [30]. Studies have shown that the longitudinal deformation of the left ventricle (LV) correlates with myocardial edema, which is detected on cardiac MRI.

Of course, MRI is the preferred method for diagnosing myocardial injury in children. This study plays a key role in assessing the structure and function of the myocardium [25,31]. According to the recommendations of the Society for Cardiovascular MRI, special MRI protocols have been developed for scanning patients with known or suspected infection caused by SARS-CoV-2 or during the period of convalescence [30]. In children with active COVID-19 or post-COVID-19, 3D sequencing is recommended to assess the coronary arteries and identify possible dilatation or ectasia of the coronary arteries in the proximal or middle segments. It is recommended to monitor blood pressure and heart rate during the study [28,32].

MRI data reported in the literature in children with myocarditis, associated with COVID-19, not different from Lake Louise criteria (LLC) myocarditis [33]. T1 (increase in myocardial relaxation time by T1, fraction of extracellular volume or late gadolinium enhancement (LGE)) with at least one criterion based on T2 (increase in myocardial relaxation by T2, visible myocardial edema, or increase in T2 signal intensity factor). Thus, to assess myocardial pathology, it is important to include T1- and T2-mapping sequences in the cardiac MRI protocol to detect myocardial changes [34,35].

However, it should be noted that there is significant variability in the criteria for diagnosing acute myocarditis associated with COVID-19, detected by MRI. Thus, the results of various studies have shown conflicting data [35]. In particular, one study described diffuse myocardial edema without signs of replacement fibrosis or focal necrosis [36,37], whereas, in another study, myocardial edema was found in 20 children in 50% of cases. Prieto et al. did not find any signs of acute myocarditis or fibrosis according to MRI, while left ventricular dysfunction of varying degrees was detected in all patients. However, it should be noted that, in this series of cases, MRI was performed after discharge, on average 16 days after admission [38]. This fact suggests that the variability of the MRI picture depends on the duration of the study.

This assumption can be supported by the results of works wherein MRI was performed to diagnose myocardial damage in children with MIS-C [39,40]. (Thus, one week after the onset of MIS-C symptoms in children admitted to the intensive care unit with signs of shock and myocarditis, diffuse myocardial edema was detected on T2-weighted images and native T1-image mapping without signs of LGE [36].) Another study found no evidence of edema on T2-weighted sequences six months after discharge, but LGE was detected in a subset of children [39]. An extended relaxation time on T1 images and no LGE in a child three months after MIS-C were demonstrated by Webster et al. [36].

There are also data in the literature on performing CT with angiography in children using a low dose of radiation. Thus, more than half of the children had ectasia of the coronary arteries, and dilatation in a quarter was examined. The detection of coronary artery lesions was higher with CT compared with echocardiography data [41]. Similar results and conclusions were obtained in other studies, according to which more than half of the cases during ECHOCG do not diagnose coronary artery aneurysms [36,42].

There are also data on positron emission tomography with 13 N-ammonia to assess reserve blood flow in the myocardium in children with heart disease associated with COVID-19. The results of these studies have shown the reliability of this technique for understanding vascular damage and predicting cardiovascular events [43,44]. However, given the radiation exposure during CT (computer tomography) and PET (positron emission tomography), in the pediatric population, the use of MRI with intravenous contrast and cardiosynchronization is preferable.

Another diagnostic method that is the gold standard and plays a key role in the diagnosis of myocarditis is endomyocardial biopsy (EMB) [45]. EMB studies have shown signs of eosinophilic myocarditis in children with myocarditis associated with SARS-CoV-2 [38], as well as interstitial edema and an increase in macrophages and T-lymphocytes [46]. However, no viral DNA was detected [38]. Another case of post-mortem histopathological examination of an 11-year-old child demonstrated the presence of myocarditis, pericarditis, and endocarditis, characterized mainly by interstitial and perivascular inflammation and foci of cardiomyocyte necrosis. The sites of inflammation included mainly CD68+ macrophages and a small number of CD45+ lymphocytes, neutrophils, and eosinophils. Electron microscopy of cardiac tissue revealed spherical viral particles 70–100 nm in diameter, corresponding in size and shape to the Coronaviridae family, in the extracellular space, as well as in cardiomyocytes, capillary and endocardial endothelial cells, macrophages, neutrophils, and fibroblasts [47].

Table 1 presents an analysis of data from studies in children during and after COVID-19.

According to the data presented in Table 1, changes in the cardiovascular system in children were noted with variability in 3.8% to 80% of cases. It should be noted that the clinical manifestations of the cardiovascular system were not confirmed by the results of ECG and ECHO ECG, which required MRI and CT diagnostics.

## 3. Morphological Changes in Myocardial Damage in COVID-19

Morphological examination is an important step in the diagnosis and confirmation of the etiology of the disease. To visualize the expression of viral antigens in various histological formations of the heart using the immunohistochemical method, monoclonal or polyclonal antibodies are used.

Considering the pantropism of many viruses, the expression of their antigens can be observed in cardiomyocytes, endothelial cells of vessels, or endocardium, as well as in inflammatory infiltrate cells, mesothelial cells of the pericardium, or in the structures of the conduction system of the heart. The detection of the expression of antigens in cardiotropic viruses in various structures of the heart, on the one hand, helps to verify the etiology of heart damage and, on the other hand, can significantly expand the understanding of the pathogenesis of virus-induced heart diseases [57].

The undoubted advantage of the immunohistochemical method is the possibility of conducting studies retrospectively in histological sections obtained from archival paraffin blocks. At present, experience has been accumulated in the verification of morphological changes using the immunohistochemical method [58].

The authors present a clinical case of the post-mortem diagnosis of heart disease in a child.

## 4. Clinical Case

A 2-year-old girl with Down syndrome, combined with atrial septal defect and hypertrophy of the right heart and primary T-cell immunodeficiency in a state of moderate severity, caused by intoxication, subfebrile fever, and clinical manifestations of lung damage. The disease had negative dynamics and, despite the ongoing treatment, on, the 17th day from the moment of hospitalization, there was a fatal outcome.

Anamnesis vitae: The girl from a second pregnancy, delivery at 37 weeks, birth weight 3100 g, height 51 cm, Apgar score 8/9 b. The neonatal period was without features.

COVID-19 appeared with rhinitis, then after three days, there was fever. On the ninth day, cough and diarrhea were registered. Symptomatic and antiviral therapy was prescribed. On the 10th day, due to negative dynamics and an increase in clinical symptoms, the child was hospitalized.

Symptoms of intoxication, fever, cough, and shortness of breath were examined. Maculopapular rash elements were noted on the body skin.

On an examination, breathing was weakened, and various rales were heard in the lungs on the right and left. The sespiratory rate was up to 30 per minute. Saturation—97%.

In the X-ray examination, bilateral polysegmental focal confluent pneumonia was revealed.

On the first day of hospitalization the negative dynamic increased against a background of antibacterial and infusion therapy. Febrile fever and shortness of breath were registed. New petechial elements appeared on the skin. According to the ECG, there was a violation of the function of the heart.

According to laboratory data, leukopenia, thrombocytopenia, an increase in ALT, AST, an increase in ferritin up to 2131 ng/mL, and hypoalbuminemia were noted. A positive PCR test result for SARS CoV-2 was obtained.

On the first day of hospitalization, the child was transferred to the intensive care unit under observation with oxygen therapy.

Infusion detoxification and antibacterial and antiviral therapy were carried out, and the negative dynamics continued.

On the fourth day, due to the progression of respiratory failure and the depression of consciousness to the level of stupor, as well as the increase in respiratory acidosis, she was transferred to mechanical ventilation. On the 15th day of COVID-19, due to the increase in the volume of infiltration and overcoming the level of markers of the “cytokine storm”, tocilizumab was administered twice at 8 mg/kg. Pentaglobin was given. Hemotransfusion was performed.

Pulmonary hemorrhage was registered a few days later. Echocardiography revealed signs of pulmonary hypertension, dilatation of the right heart chambers, and tricuspid insufficiency. She was constantly on three inotropic drugs.

On the 16th day of the hospital stay (26th day from the onset of COVID-19), the negative dynamics was examined due to the increase in the phenomena of acute heart failure and the appearance of subcutaneous emphysema of the chest. Pneumoperitoneum and pneumomediastinum were diagnosed. Drainage was performed. Resuscitation measures were started, and after 30 min, death was declared.

**Figure 1:**
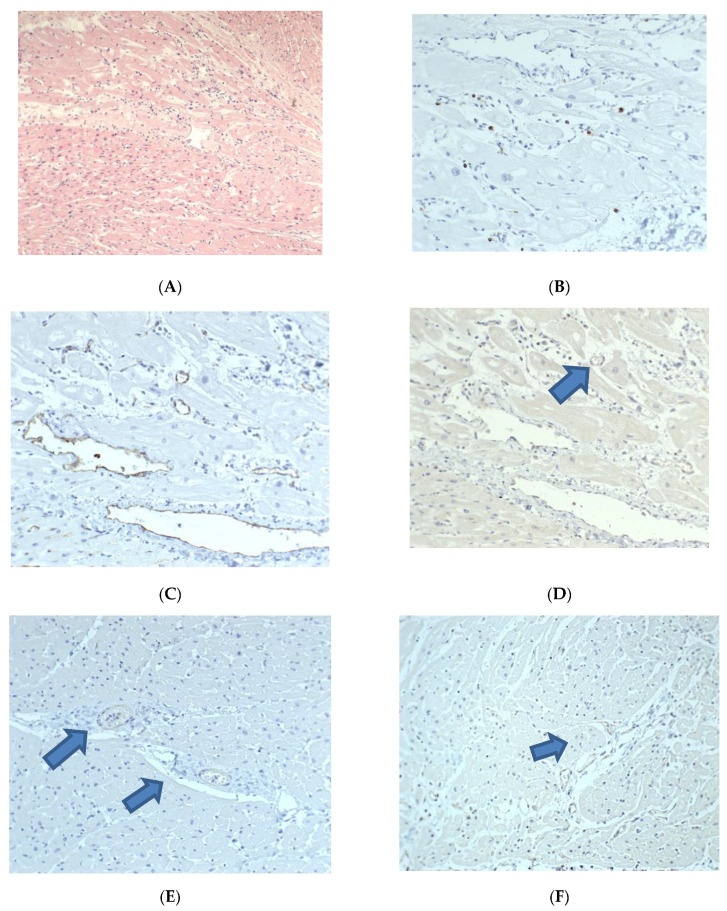
Heart changes in a 2-year-old child who died from COVID-19 (this is an original figure). Focal acute polymorphic cell myocarditis, pathological changes in intramural blood vessels, and expression of SARS-CoV2 spike antigen (brown arrow) by endothelial and smooth muscle cells of myocardial blood vessels. (**A**)—H&E, (**B**)—IHH, mouse monoclonal anti-CD45 (Thermo, USA); (**C**)—IHH, mouse monoclonal anti-CD31 (Thermo, USA); (**D**–**F**)—IHH, rabbit polyclonal anti-SARS-CoV-2 Spike (GeneTex, USA), (**A**,**B**,**D**). The length of the scale segment (**A**)—200 μm, (**B**,**C**)—100 μm; (**D**–**F**)—100 µm.

Posthumously from the autopsy material of SARS-CoV-2 nucleotide sequences by PCR in cases of acute respiratory failure caused by progressive viral pneumonia, pathological anatomical examination in the lungs revealed characteristic morphological manifestations of diffuse alveolar damage in the form of accumulations in the lumen of the alveoli, bronchioles and bronchi of fibrin; “hyaline membranes”; the thickening of the interalveolar septa due to edema and pathological cellular infiltration; manifestations of circulatory disorders; and alveolar edema, as well as the virus-induced transformation of epithelial cells (Figure 1).

In the heart, along with dystrophic changes in cardiomyocytes of varying severity, manifestations of circulatory disorders, and the formation of unevenly expressed intermuscular edema, there were small foci of polymorphocellular interstitial “aggressive” infiltration (lymphohistiocytic with a content of 10–15 infiltrating cells in the field of view at a total magnification of the microscope ×400), with the invasion of single lymphocytes within the muscle fibers with manifestations of their damage (Figure 1A,B).

Intramural blood vessels with the widespread swelling of polymorphic endothelial cells (Figure 1C), some of which expressed SARS-CoV-2. Also outside the foci of exudative inflammation, smooth muscle and endothelial cells of intramural blood vessels of the arterial type expressed SARS-CoV-2 (Figure 1E,F).

The revealed pathological changes demonstrate direct damage to the structural elements of myocardial blood vessels, aggravating its severe hypoxic changes due to respiratory failure caused by SARS-CoV-2 lung damage with the development of severe acute diffuse alveolar damage and the implementation of cell-mediated immune damage to the myocardium due to its viral damage. These changes led to the death of the child against the background of congenital heart disease and immunodeficiency.

## 5. Discussion

Differential diagnosis for myocardial damage is carried out with various viral agents, because we do not have unequivocal information that confirmed the infection of the patient with the SARS-CoV-2 virus.

The main mechanisms of myocardial injury in patients with COVID-19 include: a cytokine storm caused by an unbalanced response of T-helper 1 (TH1 cells) and T-helper 2 (TH2 cells); respiratory dysfunction and hypoxemia; and decreased activity of ACE2, which has a protective effect on the cardiovascular system as a counterregulatory element of angiotensin II signaling [58,59].

A number of studies has demonstrated that myocardial injury caused by the SARS-CoV-2 virus may be associated with increased viscosity, enhanced coagulation cascade, pro-inflammatory effects, and endothelial cell dysfunction [60]. Patients showed degeneration, hypertrophy and necrosis of cardiomyocytes, moderate interstitial hyperemia, and edema, along with the infiltration of lymphocytes, monocytes and neutrophils, but without a viral component in the myocardial tissue [54]. In addition, myocardial injury may include atherosclerotic plaque rupture, coronary vasospasm, hypoxic vascular injury, and direct endothelial or microthrombi formation [61]. Pericytes infected with the SARS-CoV-2 virus lead to the development of dysfunction in capillary endothelial cells or microvessels, followed by their necrosis [20].

On the backdrop of a large arsenal of methods for diagnosing myocardial damage, the lack of data on the most effective methods and criteria for diagnosing myocardial damage in the pediatric population during and after COVID-19 causes great difficulties [42]. Despite unprecedented collective efforts, the issues of choosing effective and sensitive diagnostic methods are still very relevant and require further research.

At present, the mechanism of the development of myocardial damage against the background of COVID-19 has not been fully elucidated. The cause of cardiocytolysis can be hypoxemia, coagulopathy, endothelial dysfunction, or the decreased production of nitric oxide [62].

The researchers note that acute coronary syndrome may be one of the initial manifestations of COVID-19 infection, which can range from ST elevation myocardial infarction to takotsubo cardiomyopathy, while ischemia and myocardial infarction may be secondary to plaque rupture caused by a stress response. or due to thrombosis [61,63].

Probably, myocarditis occurs as a result of a combination of two processes. First, local cellular dysfunction and inflammation are identified due to direct myocardial injury and the suppression of ACE2 receptors caused by viral spike proteins. A cardiotoxic catecholamine and cytokine storm develop due to a systemic hyperinflammatory response [40]. With the help of ACE2 and its assistant, transmembrane serine protease 2 (TMPRSS2), SARS-CoV-2 enters the cell [64]. The virus, causing damage to the alveoli, leads to the development of a local inflammatory reaction with the release of a large number of cytokines, including IL-6, IL-1, tumor necrosis factor α, and interferon gamma (cytokine storm), which can later develop into hyperinflammation. In addition, patients with COVID-19 have neurological, cardiovascular, intestinal, hepatic, and renal disorders [44].

However, it should be noted that, according to the EMB data in adult patients, as well as in a child with heart damage, SARS-CoV-2 virus particles were detected in macrophages, as well as in myocardial interstitial cells. This indicates the tropism of the virus to cardiomyocytes and the possibility of their direct damage [61].

It is assumed that damage to the cardiovascular system in patients with COVID-19 as a result of a systemic inflammatory response occurs due to ischemia and vasculitis with coronary artery disease. In addition, the cytokine storm and systemic inflammation caused by SARS-CoV-2 lead to hypoperfusion, overstimulation of β-adrenergic receptors, thrombosis, and thromboembolism, and lung damage and subsequent hypoxia increased due to the existing stress [11,58].

**Figure 2:**
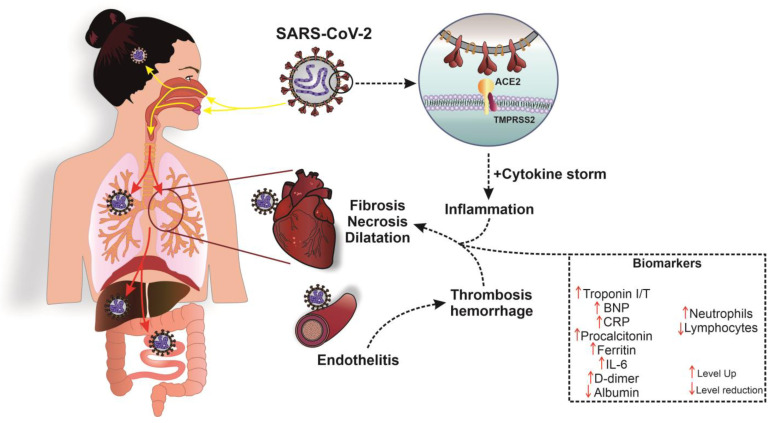
The pathogenesis of SARS-CoV-2 and its main morphological manifestations in myocardial damage. ACE2, angiotensin-converting enzyme 2; BNP, brain natriuretic peptide 32; CRP, C-reactive protein; IL-6, interleukin-6; SARS-CoV-2, severe acute respiratory syndrome-related coronavirus 2; TMPRSS2, transmembrane serine protease 2. ↑ level decrease; ↓, level increase.

The pathogenesis of heart damage against the background of COVID-19 is schematically shown in Figure 2.

Severe inflammatory processes affecting the cardiovascular system lead to the development of arrhythmias, myocarditis, cardiomyopathy, and acute heart failure. SARS-CoV-2, affecting vascular endothelial cells, disrupts their normal functioning and maintenance of tone, which further induces an increase in blood clotting and the formation of blood clots, ultimately leading to the formation of cardiac pathologies [65].

The results of fundamental and clinical studies have demonstrated the association of pro-inflammatory cytokines, mainly IL-6, with the risk of developing long QT syndrome and ventricular tachycardia [56]. It is assumed that direct myocardial damage, as well as a systemic hyperinflammatory response against the background of COVID-19, are significant proarrhythmic factors. In addition, hypoxemia and electrolyte imbalance can also cause arrhythmias [66].

Despite the fact that the defeat of the respiratory system is dominant in SARS-CoV-2 infection, the involvement of the heart in the pathological process is one of the most serious complications of COVID-19 in both adults and children. It is likely that patients with concomitant heart diseases are at high risk for the development of cardiac complications and a worse prognosis for the long-term course of post-COVID-19 in the future. Children who initially have arrhythmias, genetically determined heart pathology, or signs of heart failure may be a risk group for the severe course of COVID-19 and its consequences [63].

Undoubtedly, the use of MRI, especially in pediatrics, is popular due to the non-invasive nature of the procedure; the absence of radiation exposure; high sensitivity; and the ability to assess the function, structure and characteristics of myocardial tissue. However, the validation of MRI parameters in children and the determination of the timing and criteria for the dynamic diagnosis of acute myocarditis in children with myocarditis in COVID-19 are required [33].

It should be taken into account that the revealed histopathological changes, such as degeneration, apoptosis, necrosis of cardiomyocytes, moderate interstitial hyperemia, edema of myocardial tissue, mediated by pro-inflammatory effects and dysfunction of capillary endothelial cells damaged by the virus, are the main markers of SARS-CoV-2 [67]. These cardiovascular injuries in children often lead to the development of arrhythmias of varying severity, dysfunction of the left ventricular myocardium (systolic dysfunction), dilation of the coronary arteries, and the formation of large aneurysms of the coronary arteries.

## 6. Conclusions

Myocardial damage affected children of different ages, and some of these children had a severe form of the disease that required inotropic support and mechanical ventilation and that led to death. Based on the results of our analysis data, it is obvious that there are some difficulties in the diagnosis of SARS-CoV-2-associated myocarditis and myocarditis with other etiology. It should be known that SARS-CoV-2-associated myocarditis has higher C-reactive protein and pro-B-type N-terminal natriuretic peptide (NT-proBNP) levels in comparison with myocarditis with other etiologies.

Most often, according to EchoCG, myocardial dysfunction associated with the inflammation in coronary arteries is detected. However, the sensitivity of echocardiography in the diagnosis of SARS-CoV-2-associated myocarditis disorders was significantly lower than MRI.

The key role in cardiac MRI plays in assessing the structure and function of the myocardium, including in cases of known or unknown infection caused by SARS-CoV-2. Moreover, in SARS-CoV-2-associated myocarditis, the MRI protocol should include T1 and T2 and the mapping 3D sequence for assessing the coronary arteries in order to diagnose possible dilatation or ectasia of the coronary arteries in the proximal or middle segments.

It should be noted that the MRI picture varies, and the results of cardiac MRI are contradictory. However, in all cases, certain changes have been detected. Diffuse myocardial edema may be seen without signs of replacement fibrosis or focal necrosis, or such changes may be absent, which most likely depends on the stage of the disease during which the study is performed, but left ventricular dysfunction of varying degrees is found. This fact suggests that the variability of the MRI picture depends on the duration of the study. Furthermore, it requires the development of a unified approach to the timing of cardiac MRI and the creation of an examination protocol. The CT of the chest organs is extremely important in diagnosis, due to the fact that, according to EchoCG, coronary artery aneurysms are not diagnosed in half of patients, and their presence is confirmed only according to CT data. Moreover, more than half of the children were found to have ectasia of the coronary arteries and dilatation in a quarter of the examined [36,41,42].

At present, attempts have been made to determine highly sensitive diagnostic and prognostic methods for detecting the involvement of the heart in the pathological process, for example, an elevated troponin level, the evaluation of longitudinal ventricular strain according to echocardiography, and the modification of the MRI criteria have not been successful.

Effective methods for diagnosing heart damage during and after COVID-19 in children have not been determined yet. There is also no complete understanding of the pathophysiological mechanism underlying heart damage during SARS-CoV-2 infection. It is assumed that immune-mediated inflammation in children plays a crucial role in the involvement of the myocardium in the pathological process.

However, the direct damaging effect of the virus also contributes both to the development and aggravation of the clinical picture of the infectious process [68,69]. Long-term follow-ups are needed to fully understand the pathophysiological mechanisms and long-term prognosis in children with a cardiac injury from COVID-19 [68].

There is no doubt that studying and understanding the pathogenetic mechanisms of myocardial injury in children with COVID-19 will help in predicting and identifying patients at increased risk of heart injury, thereby accelerating their early treatment and prevention of complications.

## Figures and Tables

**Table 1:** Clinical manifestations and functional disorders of the heart in children affected by COVID-19.

№	Author Name and Year of Publication	Country	Children Examined (n)	Symptoms (n, %)	Signs of Damage to the Cardiovascular System (n, %)
1	Miller, 2021 [13]	GB	4678	n/d	Cardiac disorders have been reported (529; 11.3%).
2	Ashkenazi-Hoffnung, 2021 [14]	Israel	90	Palpitations (8; 8.9%)	Abnormal ECG changes (2; 2.2%) Pathological changes on the heart MRI (1/3; 3.3%)
3	Brackel, 2021 [48]	Netherlands	89	Palpitations (16; 17.9%)	n/d
4	Buonsenso, 2021 [38]	GB, USA	510	Palpitations 2058; 40.2%)	n/d
5	Buonsenso, 2021 [46]	Italy	129	Chest tightness (8; 6.2%) Palpitations (5; 3.8%)	n/d
6	Osmanov, 2021 [49]	Russia	518	Cardiac disorders (9/470; 2%)	n/d
8	Belay, 2021 [50]	USA	1733	Hypotension (880; 50.8%) Shock (638; 36.8%)	Myocardial dysfunction (484; 31.0%) Myocarditis (300; 17.3%) Coronary artery dilatation or aneurysm (258; 16.5%) Pericardial effusion (365, 23.4%)
9	Yasuhara, 2021 [26]	USA	917	Shock with increasing cardiac enzymes (65.8%)	Myocardial dysfunction or myocarditis (55.3%) Pericardial effusion (31.7%) Coronary artery aneurysm (21.7%)
10	Son, 2021 [27]	USA	518	n/d	Decreased LVEF less than 55% (212; 41%) Aneurysm of the coronary arteries (64; 12%)
11	Theocharis, 2021 [36]	GB	20	n/d	Decreased LV EF less than 55% (10.50%) Decrease in EF less than 55% according to MRI 35% Pericardial effusion (2; 10%) Dilatation coronary arteries (9/12; 75%) Ectasia of coronary arteries (12; 60%) Myocardial edema (10; 50%) Subendocardial infarction (1; 5%)
12	Whittaker E., 2020 [51]	GB	58	Shock (29; 50%)	Myocardial dysfunction with outcome in shock (29; 50%) Aneurysm of the coronary arteries (8; 14%)
13	Belhadjer, 2020 [52]	France Switzerland	35	Cardiogenic shock with collapse (28; 80%)	Dilatation of the coronary artery (6; 17%) Decreased EF less than 50% (35; 100%) Ventricular arrhythmia (1; 3%)
14	Toubiana, 2020 [53]	France	21	Shock syndrome (12; 57%)	Myocarditis (16; 76%) Dilatation of the coronary artery (5; 24%)
15	Feldstein, 2020 [54]	USA	186	Symptoms of heart disease (149; 80%)	Decreased EF less than 30% (9; 5%) Decreased EF 30–55% (61; 33%) Pericarditis or pericardial effusion (49; 29%)
16	Dufort, 2020 [55]	USA	99	Hypotension (32; 32%)	Myocarditis (52; 53%) Coronary artery aneurysm (9; 9%)
17	Kaushik, 2020 [56]	USA	33	Hypotension (21; 63%)	Decreased EF less than 30% (4; 12%) Decreased EF 30–55% (17; 53%) Pericardial effusion (15; 46%) Cardiomegaly (10; 30%)

## Data Availability

Not applicable.
